# Vaccine Hesitancy as a Challenge or Vaccine Confidence as an Opportunity for Childhood Immunisation in India

**DOI:** 10.1007/s40121-020-00302-9

**Published:** 2020-05-23

**Authors:** Ashish Agrawal, Shafi Kolhapure, Alberta Di Pasquale, Jayant Rai, Ashish Mathur

**Affiliations:** 1 Medical Affairs Department, GSK, Hyderabad, India; 2 Medical Affairs Department, GSK, Mumbai, India; 3 Medical Affairs Department, GSK, Wavre, Belgium; 4 Medical Affairs Department, GSK, Lucknow, India; 5 Private Practitioner, 4-Kabir Marg, Lucknow, India

**Keywords:** Immunisation, India, Vaccination, Vaccine confidence, Vaccine hesitancy

## Abstract

Vaccines have contributed substantially to decreasing the morbidity and mortality rates of many infectious diseases worldwide. Despite this achievement, an increasing number of parents have adopted hesitant behaviours towards vaccines, delaying or even refusing their administration to children. This has implications not only on individuals but also society in the form of outbreaks for e.g. measles, chicken pox, hepatitis A, etc. A review of the literature was conducted to identify the determinants of vaccine hesitancy (VH) as well as vaccine confidence and link them to challenges and opportunities associated with vaccination in India, safety concerns, doubts about the need for vaccines against uncommon diseases and suspicions towards new vaccines were identified as major vaccine-specific factors of VH. Lack of awareness and limited access to vaccination sites were often reported by hesitant parents. Lastly, socio-economic level, educational level and cultural specificities were contextual factors of VH in India. Controversies and rumours around some vaccines (e.g., human papillomavirus) have profoundly impacted the perception of the risks and benefits of vaccination. Challenges posed by traditions and cultural behaviours, geographical specificities, socio-demographic disparities, the healthcare system and vaccine-specific features are highlighted, and opportunities to improve confidence are identified. To overcome VH and promote vaccination, emphasis should be on improving communication, educating the new generation and creating awareness among the society. Tailoring immunisation programmes as per the needs of specific geographical areas or communities is also important to improve vaccine confidence.

**Fig. 1:**
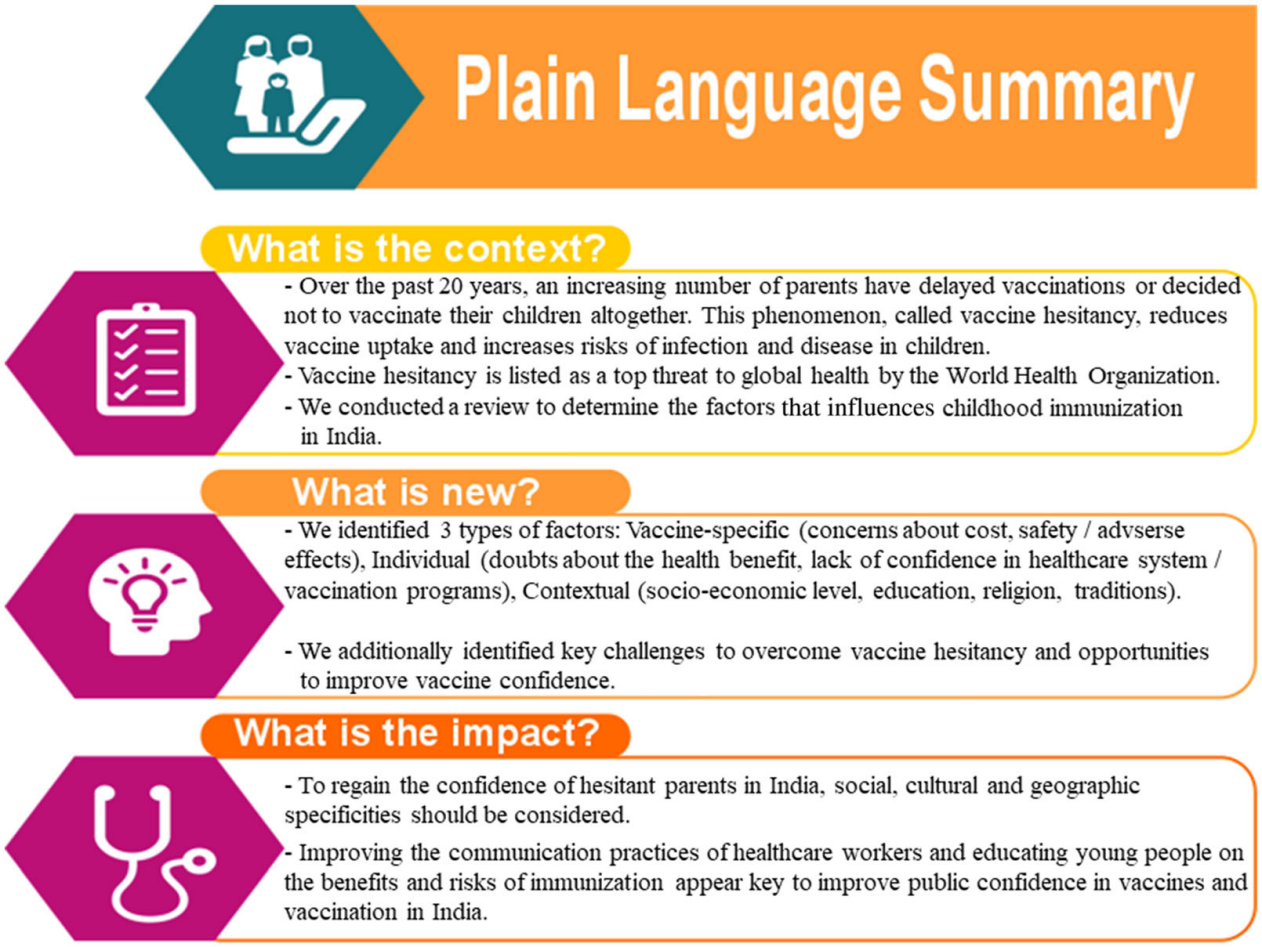
Plain language summary

Plain language summary

## Key Summary Points

**Table:** 

Rumours and controversies around vaccine safety have shed light on the strong presence of parental vaccine hesitancy (VH) in India.
A literature review identifies challenges to overcome VH as well as opportunities to improve vaccine confidence.
Vaccine-specific causes (e.g., cost, safety concerns), individual (e.g., doubts about need to vaccinate, lack of confidence in vaccination programmes) and contextual (e.g., religion, traditions) influences are involved in parental VH.
Healthcare workers and other health actors have a crucial role in improving confidence towards childhood vaccination by communicating accurate information about risks and benefits of vaccines to parents.
Educating, creating awareness and tailoring immunisation programmes for each vaccine are proposed avenues to improve parental confidence in vaccination.

## Introduction

Public concerns about vaccines are as old as vaccines themselves, ranging from safety concerns to doubts about the needs for vaccination [1]. The internet has enhanced opportunities for anti-vaccine people to connect, organise and increase their share of voice at global level. As a result, the antivaccine community has succeeded in influencing individual's behaviour and lowering confidence in vaccination despite its proven effectiveness [2].

Thus, in recent years, attention has grown around the behaviour of individuals ranging from those who are total acceptors to those who are complete refusers, i.e., hesitant to take vaccines [3]. Refusing or delaying vaccination contributes to gaps in vaccine uptake and immunisation coverage—a significant factor in controlling or eliminating vaccine preventable diseases (VPDs). Thus, vaccine hesitancy (VH) is not only a threat to elimination of VPDs (e.g., measles, polio, etc.) but is also a major factor contributing to re-emergence of such diseases. There may be multiple factors stimulating VH, depending on the context, individuals and specific features of the vaccines [3, 4]. Aside from addressing these factors, improving vaccine confidence through better communication, by health authorities as well as by the scientific and pharmaceutical communities, may help to counteract VH [5–7]. The World Health Organisation (WHO) listed VH among the top ten threats to global health in 2019 [8]. WHO defined VH as “(…) delay in acceptance or refusal of vaccines despite availability of vaccination services. Vaccine hesitancy is complex and context specific, varying across time, places and vaccines. It is influenced by factors such as complacency, convenience and confidence” [3].

India, as a country, has the largest birth cohort in the world, with 27 million children born each year. It has not been able to reach the goal of 90% coverage for all vaccines included in national immunisation schedule because of various factors including VH [9, 10]. To control VPD, it is crucial to achieve high vaccination rates—this can be achieved by countering anti-vaccination messages and by increasing public confidence in vaccines [11–13]. It also appears key to develop campaigns that address the concerns faced by individuals hesitant to vaccinate themselves or their children [14–16].

The objective of this qualitative literature review was to identify (1) the set of VH determinants impacting childhood immunisation in India, (2) key challenges to overcoming reluctance to vaccination in the country and (3) major opportunities to minimise VH in India and increase confidence around vaccination.

## Literature Search

First, a literature search was conducted in PubMed for research articles with “hesitancy” [All Fields] AND “India” [All Fields] over the 2015–2019 period and in Embase for additional articles published during the same period using the following search equation: (‘vaccine hesitancy India’ OR ((‘vaccine’/exp OR vaccine) AND hesitancy AND (‘India’/exp OR India))). Only articles reporting results from a quantitative or qualitative survey about VH in India were included. The search for VH determinants was complemented by narratives about recent vaccine-related controversies as well as a selection of challenges and opportunities relevant to the Indian context.

Furthermore, this article is based on previously conducted studies and does not contain any studies performed by any of the authors with human participants or animals.

## Determinants of VH in India

**Fig. 2:**
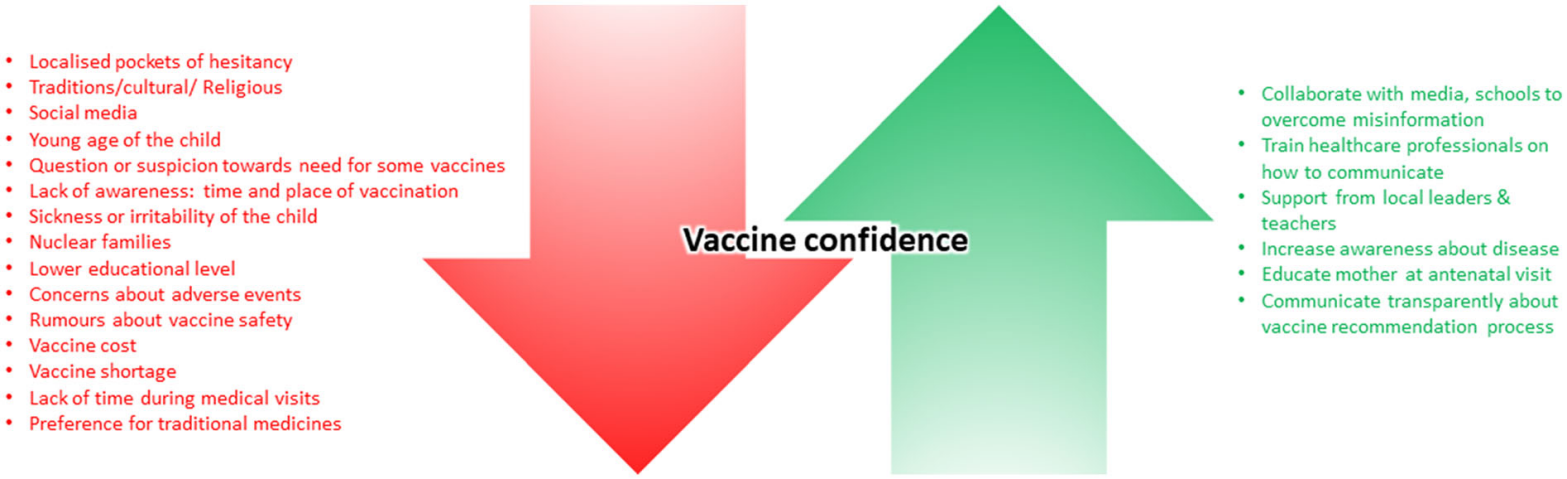
Factors negatively (red) and positively (green) influencing vaccine confidence in India

After exclusion of the articles not related to vaccination or focused on adult/traveller vaccination, nine articles reporting childhood VH determinants in India were retrieved [17–25]. Seven of them reported results from surveys or interviews of parents/caregivers in India [18–21, 23–25]. One study mixed a cross-sectional survey with interviews of parents and healthcare workers [17]. One article presented challenges reported by healthcare providers from different countries including India [22]. Among those nine articles, one reported results from the pulse polio campaign [18], and two were specific to measles-rubella (MR) vaccination [17, 19]. It was important to identify not only factors that add to VH in these articles but also factors that add to confidence in vaccination. Figure 1 elaborates on the findings in a form that can be shared with patients by healthcare professionals (HCPs) and Fig. 2 summarises the major factors that lower or improve vaccine confidence.

Social connections affect attitudes towards vaccination, as seen during the oral polio vaccination campaign [18]. Among the 1355 households with one or more children < 5 years old, 1074 (79.3%) accepted vaccination, while 137 were hesitant (10.1%) and 144 (10.6%) refused vaccination. Vaccine-refusing households had 189% more ties to other vaccine-refusing households than to vaccine-accepting households, which shows a clustering of VH communities.

Influence of social relationships and access to information through social media had an impact on the status of MR vaccination [19]. Vaccine acceptance was higher when offered at school (*P* < 0.001). It was also high among parents who trusted school teachers (*P* < 0.003) and other school children (*P* < 0.014) as sources of information. However, acceptance was lower among parents who trusted information from social media (*P* ≤ 0.036).

In another study, 14.1% of the 461 parents of children between 9 months and 15 years old were VH towards the MR campaign [17]. Mothers > 30 years old were found to be 2.65 times more prone to VH than younger ones (*P* < 0.001). Employed mothers were more prone (*P* < 0.001) to VH than unemployed mothers. VH was also more prevalent in parents with less education (*P* ≤ 0.04) than in those who had graduated. Major hindering factors were inadequate knowledge about the vaccination campaign, rumours about the safety of the vaccine, sudden planning and under-preparedness at the health system level. A major facilitating factor for the campaign was the role played by healthcare professionals in spreading awareness and increasing trust in vaccines and vaccination.

A large-scale survey (*n* = 20 749) was conducted to understand the dynamics of vaccine confidence in five countries, including India [25]. In the latter sub-group of households with children < 5 years old (*n* = 288/1 259), 36 respondents (12.5%) were found hesitant towards vaccination, among which 6 firmly refused vaccination for their children. A total of 42 reasons were provided by these 36 respondents: 13 related to confidence (safety concerns, lack of effectiveness, bad experience with vaccination or HCP or healthcare facility, preferred use of traditional medicine, religious reasons), 1 to complacency (vaccine not needed), 7 to convenience (lack of time, remoteness, vaccine shortage) and 21 to other factors (e.g., baby cries or has problems, not communicated/don’t remember). Of the 21 latter factors, “baby cries or has problem” could have been classified into “confidence”.

In another study, only 17% (33/194) of the children < 5 years old in households located in the slums of Siliguri had received vaccinations on time [23]. Reluctance to vaccinate (26.1%) and unawareness/receiving no reliable information (20.5%) were the major reasons cited for VH. Nuclear families and < 5 years schooling of the mother had higher odds of VH.

A comparison of VH across five low- and middle-income countries, including India, made using the WHO’s 10-item VH Scale, was published [21]. The VH data for India were collected between 2017 and 2018 from 309 mothers of children < 5 years old. The majority of the hesitant mothers were concerned about safety (39.2%), believed some vaccines were no longer needed (33%) or feared that newly introduced vaccines could threaten children’s health (20%). Maternal concerns about the adverse effects of vaccines, including newly introduced ones, were found at varying degrees in these five countries. No consistent association between education and VH was noted. At the same time, among all surveyed participants in Bangladesh, China, Ethiopia, Guatemala and India, a large majority perceived that vaccines are important for their child’s health (95%), that vaccines are effective (93%) and that vaccines can protect their child (94%).

In 2018, interviews at a tertiary care centre of 150 mothers of children 1 to 5 years old revealed that suspicions towards newer vaccines (61.4%), concerns about adverse events (90.7%) and perception that vaccines are not necessary for uncommon diseases (85.3%) were related to hesitant behaviour as measured by the vaccine confidence index [20, 25]. Mothers’ education was seen to protect against VH, whereas father's education, father’s use of social media and reliance on sources of information other than HCP increased the risk of VH.

A qualitative survey was conducted among 75 HCPs from four countries (UK, USA, Germany, India) in 2018 [22]. Challenges they faced were found similar (i.e., low patient-level vaccine knowledge, patient miseducation, untimely vaccine information, frequently changing schedules, pressure to achieve vaccination targets, vaccine costs). The ten Indian paediatricians interviewed reported that vaccine costs and shortages were important challenges in India. They also regret the lack of general understanding about the purpose of vaccines in segments of the population.

A questionnaire based on that created by the WHO strategic advisory group of experts on immunisation was also administered to 260 households (Balangir: 180; Nuapada: 80) in Odisha [24]. Nearly 85% had monthly incomes < 5000 Indian rupees (75 US dollars). Almost all knew that vaccines protect against infectious diseases and that parents should vaccinate their children. Around 10% highlighted long travel distances as important barriers to vaccine uptake. Nearly 28% and 9% of parents in Balangir and Nuapada, respectively, had heard negative information about the vaccines. Still, > 75% of them had their children vaccinated.

## Challenges Surrounding Vaccine Hesitancy or Confidence in India

Many challenges surround vaccination in India and need to be addressed. However, some are particularly prominent because of their impact or shared roots with broader health issues.

### Rumours and Controversies

Several controversies and false information have negatively impacted vaccine confidence over the last 20 years. During poliomyelitis vaccination programmes in early 2000, a seed of distrust was sown in particular communities by linking vaccination with sterility and by falsely claiming that pig’s blood was present in the vaccine, among other things [26, 27]. However, realising the importance of vaccination, religious leaders who were silent initially, along with community influencers, eventually actively fostered the social mobilisation that led to the successful elimination of poliomyelitis [28, 29].

Safety concerns of a severe nature were raised after the deaths of seven girls during two human papillomavirus (HPV) studies conducted in 2010 [30, 31]. An enquiry committee investigated the controversial cases and concluded that the vaccines were not responsible for the deaths [32]. Vaccination against HPV has the potential to provide great benefits for the Indian population as cervical cancer, which is mainly caused by persistent HPV infection, is the second most common cancer in Indian women [33]. Nevertheless, the call for introduction of HPV vaccine is still opposed despite recommendation by the Indian Council of Medical Research and the National Technical Advisory Group on Immunisation [34].

The decision to introduce *Haemophilus influenzae* type B (Hib) containing pentavalent vaccines in the universal immunisation programme in 2009 is yet another example of challenges regarding the need and risk-benefit ratio of vaccines. In this case, questions were raised based on studies suggesting lower burden of Hib meningitis in Indian children than in other parts of the world and based on the suggestion—that use of the pentavalent vaccines had low value to children’s health [35]. Based on available evidence, concerns were found to be unsubstantiated and the government eventually introduced the pentavalent vaccines in 2011 [35, 36].

### Social Interactions

Behaviour with respect to vaccination tends to depend on who you know, where you live or both. For example, low uptake of the MR vaccine (44% of the targeted number of children) during the 2017 vaccination campaign in Tamil Nadu [19] reflects the hesitant behaviour of parents associated with safety concerns that are usually spread through social interaction and media.

### Healthcare System and Access to Facilities

India has various geographical features with areas that are either densely or sparsely populated. In 2012, only 37% (versus 73%) and 68% (versus 92%) of people living in rural (versus urban) areas were able to access inpatient hospitalisation and outpatient facilities, respectively [37]. Results from pooled, nationally representative surveys covering 1998–2008 evidenced that lack of access to immunisation facilities, along with absence of healthcare workers, and ignorance of the place and timing for getting vaccination were among the reasons for delayed or missed vaccination [38]. Strong reductions of urban versus rural disparities in full vaccination rates from 2008 to 2013 are however noteworthy [39]. This is possibly due to the activation of primary health centres, subcentres and community health workers (Anganwadi workers) in rural areas [40]. Contrarily, precarious populations living in urban slums have been found to lack awareness about immunisation benefits and experience difficulties in accessing healthcare services [41, 42]. Lack of access to healthcare facilities and awareness are reasons for the low vaccination rates observed in the poorest strata of the Indian population [40, 42]. Lack of adequate workforce observed in both public and private sectors negatively impacts the healthcare standards and thereby the general trust of the population in the healthcare system [43]. Inadequate workforce increases the pressure on healthcare workers and may lower their availability for discussing parent’s concerns regarding vaccination. Previous negative experience with HCPs was indeed reported as one of the reasons for VH [25], and healthcare workers’ lack of empathy in slums (possibly due to elevated workloads) was perceived as a barrier in the immunisation process [44].

### Economic Factors

The community-based cross-sectional study conducted in Mumbai, one of the world's most populous cities, identified the loss of daily income as one of the most frequently reported factors for missing childhood immunisation in slum areas [44]. The cost of vaccines and vaccination is a challenge, as very few are offered free or as part of the national immunisation programme [22]. Nevertheless, VH is also observed in populations with higher socio-economic statuses and education levels [45].

### Vaccine-Specific Challenges

Vaccination schedules are designed in a way that several vaccines are administered concomitantly to improve compliance and coverage. However, due to overcrowding of vaccination schedules, HCPs and parents have concerns to administer several vaccines during a single visit [46]. Additionally, suspicions towards newly introduced vaccines, as well as doubts about the need to vaccinate against diseases that are uncommon, are recurrently reported in the Indian population [20, 21, 23].

## Opportunities to Increase Vaccine Confidence in India

Some of the above-mentioned challenges come with opportunities to address VH and increase confidence in vaccination and the health system. Moreover, identification of determinants of VH allows tailoring of immunisation programmes, campaigns and policies. Tailoring immunisation programmes has proven efficient to address gaps in vaccine uptake among population, notably by addressing VH [47–49]. Here, we present some leads that we believe are worthy to address VH in India.

### Communication

Media can have a tremendous influence on public opinion, with long-lasting impacts [50]. Today, access to social media (72.9% of households use smartphones) is far greater in India than access to TV (45.0%) or cooking gas cylinders (21.6%) [18]. Usually, mothers seek information online, especially when concerned about vaccine safety [51]. As it is difficult to control and verify all the information available on the various platforms, it is important to increase access to transparent and scientifically validated information about the risks and benefits of vaccines as well as answer questions with balanced and accurate information.

There is a global realisation that public health communicators need to adapt their communication in a way to improve trust in vaccines and vaccination [51–53]. Some proposed strategies go even one step further, suggesting to focus vaccine communication on the positive, emotional values of immunisations rather than limiting it to the scientific content [5, 6].

Healthcare workers remain the most trusted advisors among all possible sources of reliable information when it comes to vaccination [54, 55]. In that regard, and in light of the factors of VH reported in the literature search, availability and preparedness of healthcare workers for discussing vaccination are of prime importance. Some paediatricians are already engaged in improving vaccine confidence through the publication of scientifically accurate blogs with the potential to reach a broad audience [56]. The situation in low- and middle-income countries is even more closely impacted by the healthcare workers (i.e., including community health workers, Anganwadi workers, auxiliary nurse midwifes and health assistants), as they represent the frontline of vaccination and are often confronted with questions from hesitant parents [57–59]. Therefore, communication training of healthcare workers appears to be one of the most promising strategies to deal with VH and improve vaccine confidence [59]. For example, applying the CASE (Corroborate, About Me, Science, and Explain/Advise) approach could help them establish a dialogue with parents [60]. This approach could even reinforce the impact of self-help groups that are already shown to improve healthcare access and awareness in rural communities [61].

Similarly, religious leaders should also be included as important partners when communicating about immunisation as they can have a major impact when supporting it [62]. For example, the mobilisation of Muslim leaders in India was instrumental in the eradication of poliomyelitis [28].

These opportunities around communication might help overcome some of the above-mentioned challenges posed by the cultural context of India as well as by the behaviour of healthcare workers and parent’s due to safety concerns.

### Education and Creating Awareness

Educating and creating awareness about immunisation and fostering critical thinking on associated risks and benefits could have a tremendous impact on overcoming hesitant behaviour in a population [63]. Such an approach has proven advantageous in a different context, i.e., reduced use of polluting firecrackers during the Diwali festival [64, 65]. Including a basic curriculum on VPDs in schools could also have a positive impact in the long term by making the new generation aware of vaccination's risks and benefits (e.g., HPV) [66, 67]. Moreover, school teachers are recognised as a trustworthy source of information by parents accepting vaccination [19]. Schools and school teachers should therefore be engaged in vaccination campaigns as they embody knowledge to the population and also have the potential to dissipate cultural barriers, which are a deterrent to vaccination.

### Addressing Safety Concerns

Aside from their potential to mitigate parents’ safety concerns about multiple vaccine injections during a single visit, combination vaccines have economic value by reducing the number of injections and inherent administration and stocking costs [68, 69]. In India, the current schedule includes the pentavalent combination vaccine against diphtheria-pertussis-tetanus (DTP), hepatitis B (HepB), Hib and a separate polio vaccination, with either the live attenuated oral vaccine (OPV) or the inactivated injected vaccine (IPV) [70]. The use of an available hexavalent vaccine against these six diseases (DTP-HepB-Hib-IPV) could alleviate the barriers posed by parents’ fear of pain and adverse effects of vaccination. This would also foster the switch from OPV to IPV as the risk of a vaccine-derived poliovirus outbreak exists when using OPV [71, 72]. As already mentioned, the benefit of these recommendations should be transparently communicated from a public health and an economic perspectives. Assessments of the value and risk of the vaccines are important aspects for the perception of any vaccine. Therefore, nurturing and using the national surveillance programme of adverse events following immunisation is of prime importance for building evidence about vaccine safety and assuring the public that continuous monitoring is in place to help assessing any suspicion of safety issue [73].

## Summary

Immunisation has been one of the key interventions that has not only reduced morbidity or mortality of some diseases [10], but also has led to eradication of small pox and now puts polio on the verge of eradication [72, 74]. Despite these achievements, there are growing concerns with respect to vaccination acceptance among the general population, which is largely driven by lack of vaccine confidence or VH.

VH has notably been attributed to factors such as safety [17, 19–21, 23–25]; rumours and controversies (e.g., vaccination leads to infertility) [17, 26, 27]; lack of awareness about benefits of vaccines [19–25]; influence of stakeholders (e.g., local leaders) in shaping perceptions among the general population [19, 24, 26]; costs [22]; temporal and geographic barriers [23–25]; and personal attributes [17, 19, 20, 23, 25].

To address the problem of VH in India, there is a need to estimate its root causes, formulate context-specific strategies relevant to the local settings and thus help in restoring trust leading to increased confidence in vaccination. The literature revealed a diversity of settings, sometimes revealing conflicting results about the analysed factors of VH (e.g., the level of education), further highlighting the context-specific nature of VH in India. The proposed strategies include the involvement of local stakeholders; encouraging the use of different mass media techniques to increase awareness of risks and benefits and address the prevalent myths about vaccines and vaccination; improving convenience and accessibility to the vaccines; employing reminder and follow-up services; organising training sessions for healthcare workers to enhance their communication skills and ability to engage in balanced and scientifically validated dialogues with parents; providing nonfinancial incentives to immunised individuals. The success of the polio campaign that helped in its elimination in India can be attributed to all the above factors and could serve as an example for overcoming challenges in vaccination.

The issue of VH in India is vast, complex and could not be exhaustively covered in one paper. The literature search focused on VH regarding childhood vaccination resulted in the retrieval of few studies specific to it. However, we are confident that they provide useful insight into the contemporaneous concerns surrounding vaccines and vaccination in the Indian population. Also, the present literature review focused on identifying the major drivers of VH and ways to leverage these for increasing vaccine confidence.

As illustrated by the paucity of published data about VH or Confidence determinants in India and the recognised challenges surrounding vaccination, further research is needed. A first important step would be the identification of VH as well as vaccine confidence its measure across India. Survey tools could help to conduct qualitative or quantitative studies in areas of low vaccine uptake and implement strategies to increase vaccine confidence. Nevertheless, the present study provides a picture of the breadth of knowledge about VH determinants in India, identifies challenges and opportunities to improve confidence. The collective use of the identified opportunities seems key to help turn vaccines into vaccination in India.
